# Comprehensive Review of Web Servers and Bioinformatics Tools for Cancer Prognosis Analysis

**DOI:** 10.3389/fonc.2020.00068

**Published:** 2020-02-05

**Authors:** Hong Zheng, Guosen Zhang, Lu Zhang, Qiang Wang, Huimin Li, Yali Han, Longxiang Xie, Zhongyi Yan, Yongqiang Li, Yang An, Huan Dong, Wan Zhu, Xiangqian Guo

**Affiliations:** ^1^Cell Signal Transduction Laboratory, Bioinformatics Center, School of Basic Medical Sciences, School of Software, Institute of Biomedical Informatics, Henan University, Kaifeng, China; ^2^Department of Anesthesia, Stanford University, Stanford, CA, United States

**Keywords:** web server, tool, prognosis, survival, cancer

## Abstract

Prognostic biomarkers are of great significance to predict the outcome of patients with cancer, to guide the clinical treatments, to elucidate tumorigenesis mechanisms, and offer the opportunity of identifying therapeutic targets. To screen and develop prognostic biomarkers, high throughput profiling methods including gene microarray and next-generation sequencing have been widely applied and shown great success. However, due to the lack of independent validation, only very few prognostic biomarkers have been applied for clinical practice. In order to cross-validate the reliability of potential prognostic biomarkers, some groups have collected the omics datasets (i.e., epigenetics/transcriptome/proteome) with relative follow-up data (such as OS/DSS/PFS) of clinical samples from different cohorts, and developed the easy-to-use online bioinformatics tools and web servers to assist the biomarker screening and validation. These tools and web servers provide great convenience for the development of prognostic biomarkers, for the study of molecular mechanisms of tumorigenesis and progression, and even for the discovery of important therapeutic targets. Aim to help researchers to get a quick learning and understand the function of these tools, the current review delves into the introduction of the usage, characteristics and algorithms of tools, and web servers, such as LOGpc, KM plotter, GEPIA, TCPA, OncoLnc, PrognoScan, MethSurv, SurvExpress, UALCAN, etc., and further help researchers to select more suitable tools for their own research. In addition, all the tools introduced in this review can be reached at http://bioinfo.henu.edu.cn/WebServiceList.html.

## Introduction

The prognosis estimation of tumor patient is of great significance to guide clinical treatments and facilitate the elucidation of tumorigenesis mechanism. In current clinical practice, prognosis is determined by many factors, such as disease stage, clinical performance, treatment experience and understanding of the cancer development. However, these properties are relative subjective and may lead to inaccurate prognostic estimates, and may even lead to inappropriate anticancer management strategy. Genotype-Tissue Expression (GTEx) and the Cancer Genome Atlas (TCGA) projects offer a large number of RNA sequence data of normal and cancer samples, providing unprecedented opportunities for many fields such as cancer bioinformatics and precision medicine to improve our understanding in cancer development and treatment (1, 2). Molecular prognostic biomarkers are the basic components of precision medicine. Data mining and other biological analysis make it possible to predict the prognosis of tumors at the molecular level (3–5). Accurate clinical estimation using prognostic biomarkers helps determining optimal anti-cancer treatment. At the same time, it provides assistance in developing more detailed hospice care plans. So in recent years, the discovery of prognostic biomarkers has become a hot topic in precision medicine.

Numerous studies have evidenced that molecular markers in DNA, RNA and protein level can be as prognostic biomarkers in cancer, and guide the effect of treatment either independently or in addition with present prognosis systems (6–8). In these study, Kaplan-Meier method and multivariate Cox proportional hazards regression models were commonly used to evaluate the associations between molecular markers and survival of patients with cancer (9, 10). However, these biomarkers are not suitable for clinical application due to the lack of independent validation and poor repeatability between different studies.

Mining data from public datasets and making assessments and predictions can be challenging and time-consuming. To extract useful information from these datasets, it requires researchers with strong bioinformatics expertise. To allow more researchers be able to quickly extract information they need, online tools that can easily perform survival analysis from these data are needed. The rapid growth of public datasets has enabled some research groups to focus on collecting omics datasets and developing online bioinformatics prognostic tools and web servers. These various prognostic analysis tools provide valuable evidence and ideas for cancer researchers. However, for many researchers and clinicians, it may be difficult to find the most suitable tool for their own research quickly. This review attempts to provide a comprehensive overview of the commonly used online prognostic tools for cancer prognostic analysis. In addition, the main challenges and future directions in this field are also discussed in this paper.

## Materials and Methods

**Figure 1:**
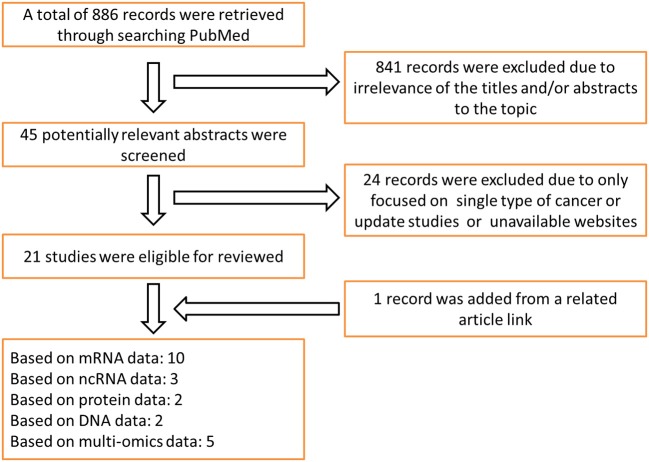
Search flowchart: prognostic web servers for cancers included and excluded in each step.

**Figure 2:**
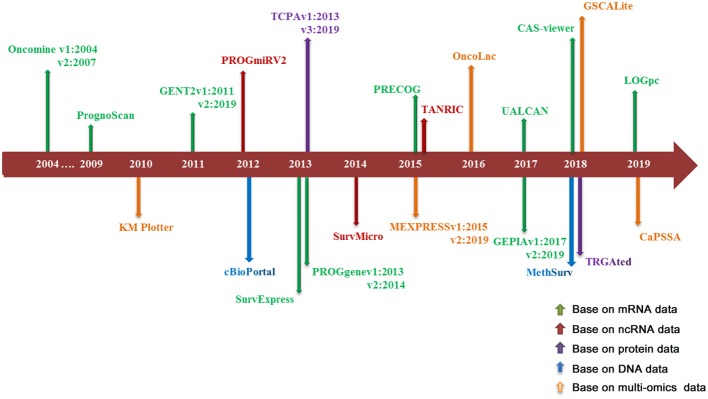
The time axis for the publication of prognostic web servers.

Literature research and data collection: the survival analysis tools reviewed in this paper include online prognostic bioinformatics tools and web servers developed by applying different types of profiling data (genomics, epigenomics, proteomics etc.) from clinical samples of different cohorts. Search Strategy for prognostic tools was executed in PubMed and Google Scholar from Jan 1, 2000 to August 31, 2019. Search terms include: “survival analysis,” “web server,” “prognostic biomarker” and “cancer,” keywords combination was used for search. The search was limited to English language. There are 886 articles that matched to above criteria. In the review, 22 representative databases that can be used for the prognosis analysis of multiple cancer types were selected for detailed description; because most of the prognostic tools for single type of cancer were included in the above databases, so we just gave a brief introduction. Ten of these databases are based on mRNA profiling data for prognostic analysis, three databases based on ncRNA profiling data, two databases based on protein data, two databases based on DNA data, and five databases based on multi-omics data. The literature retrieval process is shown in Figure 1. The release time of prognostic databases is presented in Figure 2. The date of the last search and collating data for these databases was December 10, 2019.

## Results

### Web Servers for Survival Analysis Based on mRNA Data

In the past two decades, high-throughput gene chips and next-generation sequencing technologies have provided opportunities to explore important cancer-related molecules, therapeutic targets, diagnostic, and prognostic biomarkers. With the implementation of the Cancer Genome Atlas (TCGA) project, a large number of epigenome, transcriptome, and proteome data of tumor samples became publicly accessible. Researchers can analyze the correlation between these data and survival, and look for prognostic biomarkers. Many studies have shown that mRNA expression is closely related to cancer prognosis (11–13). In order to promote the development and evaluation of prognostic biomarkers, some research groups have developed prognosis tools and web servers based on mRNA data by mining TCGA and GEO (Gene Expression Omnibus) data and adding complex statistical calculation. This review introduces 14 bioinformatics tools for evaluating cancer prognosis based on mRNA data (Table 1).

**Table 1:** Comparison of prognostic web servers based on mRNA data.

**Web server**	**Datasets**	**Cancer types**	**Samples**	**Subgroup** **analysis**	**Multi-gene query**	**Optimal cut-off**	**Login required**
LOGpc	193	26	28,098	Yes	No	No	No
GENT2	195	27	–	Yes	No	No	No
PROGgeneV2	193	27	28,503	Yes	Yes	No	No
SurvExpress	144	26	29,110	Yes	Yes	No	No
PRECOG	165	39	19,168	Yes	No	No	Yes
Oncomine	103	25	17,217	Yes	No	No	Yes
PrognoScan	74	23	9,196	No	No	Yes	No
KM Plotter	45	21	12,984	Yes	Yes	Yes	No
GSCALite	63	33	10,558	Yes	Yes	No	No
UALCAN	35	31	7,233	Yes	Yes	No	No
GEPIA	33	33	10,558	No	Yes	No	No
CAS-viewer	33	33	10,558	Yes	No	No	No
MEXPRESS	33	33	–	Yes	No	No	No
CaPSSA	28	27	10,206	No	Yes	No	No
OncoLnc	21	21	8,616	No	No	No	No

–, *survival sample data is not displayed on the website*.

#### LOGpc^1^

LOGpc is a web server that contains a large number of datasets for survival analysis, which provides 13 types of survival terms for 28,098 cancer patients from 26 types of malignant tumors, including OSlms, OSblca, OSkirc and other 23 online prognostic tools (14–21). These patient samples were collected mainly from TCGA and GEO cohorts. LOGpc is free and easy to operate. Twenty six types of tumors are classified into 11 system categories according to TCGA. Currently, only official gene symbol input is acceptable in LOGpc. When user input the gene symbol and set the relative parameters, then click on the “Kaplan-Meier plot” button and the results will be displayed on the output webpage. In order to meet the specific needs from different researchers, clinical confounding factors can also be defined for advanced subgroup analysis.

#### GENT2^2^

GENT2 provides the differential expression analysis and prognosis analysis based on tumor subtypes (22). The users can search the gene expression profiles of different tissues, and compare the expression levels between tissue subtypes. For survival analysis, this tool provides Kaplan Meier plot with log rank test and establishing Cox proportional risk model for meta-analysis. At present, it provides survival analysis for 27 cancer types, including 46 subtypes of 19 cancer types.

#### PROGgeneV2^3^

PROGgeneV2 is a web-based tool for studying the prognosis of genes in a variety of cancers (23, 24). In current it comprises 193 datasets for 27 cancer types. The users can perform survival analysis of single gene, multi genes and two genes expression ratio, and also use the function of adjusting covariate survival model. Users can upload customized gene datasets for survival analysis of interested genes and compare the results with previously published studies.

#### SurvExpress^4^

SurvExpress is for studying risk assessment and survival analysis. It contains more than 29,000 samples of 26 cancer types with clinical information from 144 datasets (25). The outputs generated by SurvExpress include the Kaplan-Meier plots by risk group, a heat map of gene expression values and a visual association of available clinical information to risk groups. Survival ROC estimates the specificity and time-dependent sensitivity for survival risk groups.

#### PRECOG^5^

PRECOG is a system for integrating genomic profiles and cancer clinical data, it covers 39 different cancer types, including about 19,000 samples with overall survival data from 165 cancer expression datasets (26). It allows researchers to query whether gene expression correlates with patient survival. For simple display, 39 different histologic types of tumors were divided into 18 groups. The correlation between gene expression and overall survival was assessed by univariate Cox regression. PRECOG also provides gene prognosis analysis for pan-cancer. However, new users need to register and log in.

#### Oncomine^6^

Oncomine is a cancer gene chip database and integrated data mining platform, aiming at mining cancer gene information (27, 28). Oncomine has more complete cancer mutation spectrum, gene expression data and related clinical information, which provides insights to identify new biomarkers or new therapeutic targets. With Oncomine, users can get the results of differential expression, co-expression analysis, molecular concepts analysis, interaction network, correlation analysis between gene expression and survival status, but Kaplan-Meier plot isn't displayed directly. Meta-analysis can also be used to compare various studies to determine more reliable and consistent results. Oncomine Research Edition is free, but needs a valid academic email address to register and log in.

#### PrognoScan^7^

Prognoscan is a platform for predicting the relationship between gene expression and patient survival based on a large number of public cancer microarray datasets with clinical information. It provides a variety of survival terms for 14 cancer types (29). One of its advantages is that survival analysis in this tool performs the minimum *P*-value method and optimal cut-off is provided.

#### KMplotter^8^

The Kaplan Meier plotter (KMplotter) can be used for single gene or multiple gene prognosis analysis for many kinds of malignant tumors (30–32). Researchers can assess the effect of mRNA and miRNA expression on the survival rate of 21 cancer types by pan-cancer analysis. When the users input the relevant gene name and select the appropriate gene expression cut-off point, the comparison results between the two groups will be displayed with 95% confidence interval, risk ratio and log rank *P*-value. An Auto best cut-off is provided to compute all possible cut-off values to get the best performing threshold in survival analysis.

#### GSCALite^9^

GSCALite is a tool for analyzing expression/variation/ clinical correlation of gene sets in cancers with dynamic and visualization manner (33). It provides three survival analysis modules for a gene set based on cancer multi-omics data of TCGA. (1) Differential mRNA expression of gene set between tumor and matched normal samples, gene expression between subtypes of each selected cancer, and its effect on overall survival rate. (2) The influence of SNV (single nucleotide variants) frequency and mutation type of gene set on the overall survival rate in a cancer type. (3) Differential expression of methylation between tumor and matched normal samples, and the effect on the survival rate of selected cancer types. It allows users to search for prognostic markers at transcriptome level, epigenetic modification, and DNA mutation. Users can query the cancer pathway activity related to gene expression and the correlation between genes and drug sensitivity, it is convenient for researchers to study drug resistance of tumor.

#### UALCAN^10^

UALCAN is a web-based tool for analyzing TCGA RNA-seq and clinical data to evaluate the association of gene expression and patient survival, allows users to conduct differential expression analysis and survival analysis for interested genes and access the expression and survival information of a given gene in 31 types of cancers by performing pan-cancer analysis (34). Currently, UALCAN provides protein differential expression analysis for breast cancer, colon cancer, and other three cancer types, but does not provide survival analysis based on protein data. UALCAN also provides additional information about the selected genes or targets by linking to Pubmed, TargetScan, DRUGBANK, and so on, this helps researchers collect more valuable information and data.

#### GEPIA^11^

GEPIA is an interactive web-based tool for survival analysis based on gene expression, it offer the choice of selecting overall survival (OS) or disease-free survival (DFS) for the analysis (35, 36). According to the characteristics of gene normalization, GEPIA allows two different genes to be input at the same time for survival analysis. GEPIA also presents the top genes most related to the survival of cancer patients. This function is very helpful for the users. In addition to providing patient survival analysis, GEPIA has other functions such as differential expression analysis based between different cancer types, multiple gene comparison, similar genes detection.

#### CAS-Viewer^12^

CAS-viewer is a web-based tool for multiple level comprehensive analysis by integrating multi-omics data such as mRNA, miRNA, methylation, SNP, and clinical information across different cancer types (37). It links the differential transcriptional expression rate with methylation, miRNA, and splicing regulatory elements of 33 cancer types. “Clinical correlation” module presents Kaplan Meier plot showing the correlation between PSI (percent spliced in) value and survival rate, and in this way users can identify potential transcripts related to different survival outcomes of each cancer type.

#### MEXPRESS^13^

MEXPRESS is an intuitive web tool for analysis of gene expression, DNA methylation, and association with clinical information including patient survival (38). It provides a very different visual interface, allows users to compare specific genomic features (such as DNA methylation) with gene expression and clinical information. Researchers can study the relationship between DNA methylation and gene expression and multiple clinical variables by using MEXPRESS platform.

#### CaPSSA^14^

CaPSSA supports users to detect the prognostic value of patient subgroups based on gene expression, mutation or genomic alterations of query genes (39). Importantly, it also supports custom histochemical data analysis with clinical information. For candidate gene sets that user-supplied, interactive patient stratification is supported based on gene expression profiles and genomic alterations, the results of log-rank test and Kaplan Meier plots will be displayed for evaluating the prognostic value.

### Web Servers for Studying Prognostic Implications of ncRNA

In the past decade, a large number of studies have shown that non-coding RNA (ncRNA) plays an increasingly important role in epigenetic regulation. ncRNAs involved in the network can affect many molecular targets which are related to the development of cancer, and many ncRNAs are considered as driving factors or suppressors of carcinogenesis (40). MicroRNA (miRNA) as one type of ncRNAs regulates mRNA at the transcriptional or post-transcriptional level (41). Studies have shown that lncRNA (long non-coding RNA) plays an important role in many life activities such as dose compensation effect, epigenetic regulation, cell cycle and cell differentiation, and has become a hot spot in tumor genetics research (42). Their expression in cancer has been studied by high-throughput methods, generating valuable sources of public available datasets. An important step in developing ncRNA biomarkers is to evaluate them in independent cohorts. To help and simplify the assessment of ncRNA signatures in cancer prognosis, several ncRNA prognostic databases have been developed by some research teams using public profiling data (Table 2).

**Table 2:** Summary of prognostic web servers based on ncRNA data.

**Web server**	**Datasets**	**Cancer types**	**Samples**	**Subgroup** **analysis**	**Biomarker**	**Multi-gene query**	**Optimal** **cut-off**	**Login** **required**
PROGmiRV2	134	33	19,025	Yes	miRNA	Yes	No	No
SurvMicro	43	15	6,412	Yes	miRNA	No	No	No
KM Plotter	25	21	10,613	Yes	miRNA	Yes	Yes	No
OncoLnc	21	21	8,648	No	miRNA	No	No	No
TANRIC	23	20	6,763	Yes	LncRNA	–	–	No
OncoLnc	18	18	8,023	No	LncRNA	No	No	No

–*, related information is not displayed on the website*.

#### PROGmiRV2^15^

PROGmiRV2 is a pan-cancer miRNA prognostics database, whose miRNA data comes from GEO and TCGA (43). Compared with version 1, the datasets and samples of the new version have increased greatly, prognosis analysis has been improved from single cancer type analysis to pan-cancer analysis, and the survival indicators provided have increased from one to three (overall survival, recurrence free survival, and metastasis free survival). Users are also allowed to upload their own customized dataset for prognosis analysis, but registration and login are required.

#### SurvMicro^16^

SurvMicro is a bioinformatics tool for analyzing cancer prognosis based on miRNA. Its data comes from GEO, TCGA, and ArrayExpress (44). SurvMicro comprises 43 datasets and more than 6,000 samples in 15 different cancer types. Cox multiple fitting was used to evaluate the risk of prognosis, the prognosis index was obtained by calculating the sum of miRNA expression value and Cox coefficients. According to the ranking of prognosis index, users would know the risk group of poor prognosis.

#### OncoLnc^17^

OncoLnc is an interactive tool for studying survival correlations for lncRNA, miRNA, and mRNA (45, 46). OncoLnc contains patient survival data of 21 cancer types from TCGA mRNAs, miRNAs, and MiTranscriptome data. The users can divide patients into subgroups according to gene expression levels, measure the result between subgroups. OncoLnc allows users to view the results of Kaplan Meier plots of one or multiple types of cancers at one time, provide Cox regression results, and download the full data used in the analysis. It also allows users to explore the survival relevance of inquired genes in 21 types of cancers at one time, this function is helpful to study whether specific genes play important roles in cancer prognosis.

#### TANRIC^18^

TANRIC is an interactive platform for multiple analysis of lncRNA in cancer (47). It includes the expression profile of lncRNA in more than 6,000 patient samples of 20 cancer types from TCGA and other three independent datasets. TANRIC consists of six modules, users can get the annotation data of lncRNA through module “My lncRNA,” and analyze whether lncRNA is related to the survival time of patients (including subtypes prognosis analysis). Users can also use other functions TANRIC to recognize the differential expression of lncRNA in tumor and normal tissue, as well as in tumor subtype or tumor stage, evaluate the differential expression of lncRNA in wild type and gene mutation cancer, evaluate the influence of lncRNA expression on drug sensitivity, and find some signal pathways related to cancer subtype defined by lncRNA.

### Web Servers for Survival Analysis Based on Protein Data

Functional proteomics is a powerful way to understand the pathophysiological mechanism and find the therapeutic target of cancer. In order to find biomarkers for prognosis and targets for treatment improvement, it is necessary to study the correlation between protein and survival. As a part of the Cancer Genome Atlas (TCGA) Project and other works, reverse-phase protein array (RPPA) was used to measure the protein expression in a large number of clinical cancer samples and cell lines (48, 49). This technology provides a necessary condition for the establishment of repeatable prediction model and protein prediction database. Here, we introduce two protein survival analysis databases based on RPPA data (Table 3).

**Table 3:** Comparison of prognostic web servers based on protein data.

**Web server**	**Datasets**	**Cancer types**	**Samples**	**Proteins**	**Subgroups**	**Multi-gene query**	**Optimal** **cut-off**	**Login required**
TCPAv3.0	35	33	8,328	258	No	No	No	No
TRGAted	31	31	7,843	245	Yes	Yes	Yes	No

#### TCPAv3.0^19^

TCPAv3.0 is an updated version of TCPA to explore and analyze protein expression based on TCGA RPPA data (50, 51). It integrates protein data and other TCGA data (somatic mutations, SCNAs, DNA methylation, mRNA and miRNA expression, and patient clinical information) and gives comprehensive protein-centric analyses. The users can find protein markers or pathway events that are significantly related to patient survival by using Cox proportional risk model and log rank test. The users can identify which proteins associated with the prognosis of different cancers and subtypes by pan-cancer analysis. The pan-cancer analysis module using multi-omic TCGA data provides researchers a unique way to validate specific protein-driven multi-omic hypotheses in multiple cancer types.

#### TRGAted^20^

TRGAted is an intuitive tool for analyzing the correlation between more than 200 proteins and survivals in 31 types of cancers (52). RPPA data (Level 4) contained in TRGAted come from the TCPA Portal. The cancer clinical information provided are comprehensive, including: gender, age, tumor stage, histological type, response to treatment. Users can use Cox proportional hazard model to analyze the prognosis of all proteins in each cancer type, or for a single protein across all cancer types. Comparison with TCPAv3.0, TRGAted provides more survival indicators, and its function of visualizing all proteins in a cancer type can help researchers find survival related proteins in the specific cancer more easily. The users are allowed to download and modify TRGAted for better usability under GPLv3 (GNU General Public License v3.0).

### Web Servers for Prognosis Analysis Based on DNA Data

Patients with genetic mutations in tumor cells are more likely to display poor pathological features, resulting in significantly altered overall survival (53). The new generation of sequencing technology has accelerated the study of somatic genetics, identifying patient subgroups with different genomic alteration patterns could facilitate to stratify patients with different clinical outcomes and to propose putative biomarkers. In addition to DNA mutation, DNA methylation is the most studied epigenetic modification which is crucial for facilitating vital biological processes such as embryonic development, genomic imprinting, and X-chromosome inactivation. Aberrant DNA methylation may lead to changes in cellular micro-environment, affect the gene expression pattern, and ultimately result in various pathological conditions including carcinogenesis (54, 55). Several recently developed high-throughput techniques facilitate genome-wide DNA methylation profiling. Some prognostic tools were also developed to facilitate the evaluation of the prognostic properties of CpG methylation data (Table 4).

**Table 4:** Summary of prognosis web servers based on DNA data.

**Web server**	**Datasets**	**Cancer types**	**Samples**	**Data types**	**Subgroups**	**Optimal** **cut-off**	**Login required**
GSCALite	33	33	10,943	Methylation	Yes	No	No
MEXPRESS	33	33	–	Methylation	Yes	No	No
MethSurv	25	25	7,358	Methylation	No	Yes	No
cBioPortal	>100	32	–	Mutation/ CNA	Yes	–	No
GSCALite	33	33	11,124	Mutation	Yes	–	No
CaPSSA	27	26	10,758	Mutation	No	–	No

–*, related information is not displayed on the website*.

#### MethSurv^21^

MethSurv is a web tool dedicating for survival analysis based on DNA methylation data including 7,358 samples in 25 different cancer types from TCGA (56). Methsurv provides multiple survival terms analysis, and the home page contains the following modules: single CpG, region based analysis, all cancers, top biomarkers, and gene visualization. Users can retrieve CpG survival analysis results of selected areas of a chromosome, and also search for a gene of interest to explore the survival statistics of all CpGs available. Users can see top biomarkers arranged according to *p*-value of all CpG labeled cancer types in the whole genome. In brief, MethSurv is a valuable platform for preliminary screening of methylation cancer biomarkers.

#### cBioPortal^22^

cBioPortal provides a visual tool for interactive exploration of multiple cancer genomic datasets (57, 58). It integrates and simplifies the data including somatic mutation, mRNA and microRNA expression, DNA copy-number alterations(CNAs) and methylation, protein, and phosphoprotein RPPA data, so that the users can obtain graphical summaries of large-scale cancer genomic data intuitively. It enables users to inquiry survival analysis based on DNA mutation data and CNA data, the results of OS, and DFS of patients are presented intuitively in the form of Kaplan-Meier plots. Pan-cancer analysis is also allowed.

### Prognostic Tools for Single Type of Cancer

Through literature search, 11 prognostic tools for single type of cancer were found (Table 5). MiRpower is a part of KMplotter database to analyze the prognostic relevance of miRNAs in breast cancer (31). OSlms, OSescc, OSkirc, OSblca, OScc, OSbrca, OSacc, and OSuvm are bioinformatics tools included in the LOGpc platform for survival analysis of leiomyosarcoma, esophageal squamous cell carcinoma, kidney renal clear cell carcinoma, bladder cancer, cervical cancer, breast cancer, adrenocortical carcinoma, and uveal melanoma (14–21). OvMark and BreastMark are online web servers for prognosis analysis of ovarian cancer and breast cancer, users can detect the prognostic potential of about 17,000 genes and 341 miRNAs in ovarian cancer and breast cancer (59, 60).

**Table 5:** Prognostic tools for single type of cancer.

**Cancer type**	**Database**	**Website**	**Data type**	**Reference**
Breast cancer	miRpower	http://kmplot.com/mirpower	miRNA	(31)
	BreastMark	http://glados.ucd.ie/BreastMark/index.html	mRNA, miRNA	(60)
	OSbrca	http://bioinfo.henu.edu.cn/BRCA/BRCAList.jsp	mRNA	(19)
Bladder cancer	OSblca	http://bioinfo.henu.edu.cn/BLCA/BLCAList.jsp	mRNA	(17)
Leiomyosarcoma	OSlms	http://bioinfo.henu.edu.cn/LMS/LMSList.jsp	mRNA	(14)
ESCC	OSescc	http://bioinfo.henu.edu.cn/DBList.jsp	mRNA	(15)
KIRC	OSkirc	http://bioinfo.henu.edu.cn/KIRC/KIRCList.jsp	mRNA	(16)
Cervical cancer	OScc	http://bioinfo.henu.edu.cn/CESC/CESCList.jsp	mRNA	(18)
Adrenocortical carcinoma	OSacc	http://bioinfo.henu.edu.cn/ACC/ACCList.jsp	mRNA	(20)
Uveal melanoma	OSuvm	http://bioinfo.henu.edu.cn/UVM/UVMList.jsp	mRNA	(21)
Ovarian cancer	OvMark	http://glados.ucd.ie/OvMark/index.html	mRNA, miRNA	(59)

## Discussion

The development of public databases (such as TCGA and GEO) provides a large number of genomic, epigenomic, transcriptional and proteomic data, and provides the possibility for gene function analysis and biological mechanism discussion (1, 2). The rapid growth of multi-omics data provides more opportunities for the research of cancer molecular mechanism and biological target, but for the researchers without strong computing power and bioinformatics background, they might face many difficulties and challenges in data mining and analysis. Since the EAPC (European Association for Palliative Care) made recommendations for the development of cancer prognostic tools in 2005, a number of prognostic tools have been developed, evolved, and validated (61). In this review, we summarized 22 prognostic bioinformatics tools, which provide survival analysis or with other functions. We analyzed and compared their key information and characteristics, follow-up information for each tool is presented in Table 6, strength and limitation are displayed in additional files (Table S1). With these tools, researchers can easily explore a large number of datasets from complex data platform, find genes, ncRNAs, proteins, gene modifications, or mutations associated with patient survival, ask specific questions and test their hypotheses (48, 62, 63). Comprehensive expression analysis can be carried out by simple clicks, which greatly promotes data mining in research fields, scientific discussions and treatment discovery processes. These tools have the potentials to integrate and personalize the prognostic information for individual patients and provide refined risk estimates for uncertain clinical management scenarios. Meanwhile each database has its own strengths. Some databases focus on survival analysis by collecting datasets of various cancer types, such as LOGpc, PROGgeneV2, KM Plotter, PrognoScan, TRGAted. Some databases provide other functions, UALCAN, and GEPIA have the function of top differential gene display, which provide a way for clinicians and researchers to select possible target genes for diagnosis or treatment, Oncomine, and TCPA provide multidimensional analysis and comparison of datas. GSCALite, TANRIC can be used for drug screening and treatment options by analyzing the correlation between therapeutic targets and lncRNAs. Advances in genome technology and computational biology provide us with an unprecedented opportunity to understand molecular events associated with cancer, and to apply precise cancer treatment. We hope this review will be helpful to clinicians and oncologists who are interested in finding prognostic or predictive features of cancer.

**Table 6:** Follow-up information of prognostic web servers.

**Web server**	**OS**	**DFS**	**RFS**	**MFS**	**PFS**	**DSS**	**Others**	**Total**
LOGpc	°	°	°	°	°	°	DFI, PFI, DMFS, DRFS,LMFS, BMFS, EFS	13
GENT2	°	°			°	°		4
PROGgeneV2	°		°	°				3
SurvExpress	°		°	°				3
PRECOG	°					°		2
Oncomine	°							1
PrognoScan	°	°	°		°	°	EFS, DMFS, DRFS	8
KM Plotter	°		°		°	°	DMFS, PPS, FP	7
GSCALite	°							1
UALCAN	°							1
GEPIA	°	°						2
CAS-viewer	°							1
MEXPRESS	°							1
CaPSSA	°	°						2
OncoLnc	°							1
PROGmiRV2	°		°	°				3
SurvMicro	°							1
TANRIC	°							1
TCPAv3.0	°				°			2
TRGAted	°					°	DFI, PFI	4
MethSurv	°							1
cBioPortal	°	°						2

“*°”, Yes; OS, overall survival; DFS, disease free survival; RFS, relapse free survival; MFS, metastasis free survival; PFS, progression free survival; DSS, disease specific survival; DMFS, distant metastasis free survival; PFI, progression free interval; DFI, disease-free interval; PFI, progression free interval; EFS, event free survival; LMFS, lung metastasis free survival; BMFS, brain metastasis free survival; DRFS, distant relapse free survival; FP, first progression; PPS, post progression survival*.

## Limitation and Prospective

**Figure 3:**
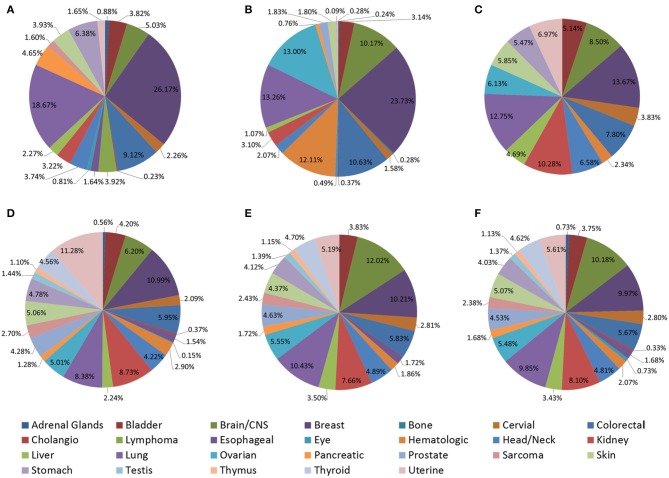
Distribution of cancer types in web servers. **(A)** LOGpc (mRNA level); **(B)** PROGmiRV2 (miRNA level); **(C)** OncoLnc (lncRNA level); **(D)** CaPSSA (mutation level); **(E)** GSCALite (methylation level); **(F)** TCPAv3.0 (protein level).

Although these tools provide great convenience for prognostic biomarker development, several key aspects of these prognostic tools remain elusive. Differences in datasets collected and split points may result in significantly different results, so we collected datasets and their source of these web servers (Figure 3 and Tables S2–S5) and found excluding TCGA data, there are significant differences in other data sources. This may be one of the reasons why the analysis results of different tools are not completely consistent. In the future, efforts should be made in data optimization, prognostic tools should be improved to be able to predict multi-gene markers, select optimal cut-off computation, use hierarchical clustering and consider complex multi-omics networks of interactions. In addition more molecular subtypes and clinical information including tumor tissue image and treatment data should be collected and mined to identify more meaningful prognostic markers through more detailed subtype analysis.

## Author Contributions

HZ, GZ, LZ, QW, and XG collected data, set up web pages, and drafted the paper. HL, YH, LX, ZY, YL, YA, HD, and WZ contributed to critical revision of the manuscript for intellectual content. All authors edited and approved the final manuscript.

### Conflict of Interest

The authors declare that the research was conducted in the absence of any commercial or financial relationships that could be construed as a potential conflict of interest.

## Footnotes

^1^http://bioinfo.henu.edu.cn/DatabaseList.jsp

^2^http://gent2.appex.kr

^3^http://genomics.jefferson.edu/proggene/

^4^http://bioinformatica.mty.itesm.mx:8080/Biomatec/SurvivaX.jsp

^5^https://precog.stanford.edu/

^6^http://www.oncomine.org/

^7^http://www.prognoscan.org/

^8^http://kmplot.com/analysis/

^9^http://bioinfo.life.hust.edu.cn/web/GSCALite/

^10^http://ualcan.path.uab.edu/index.html

^11^http://gepia.cancer-pku.cn/

^12^http://genomics.chpc.utah.edu/cas/

^13^https://mexpress.be

^14^http://capssa.ewha.ac.kr

^15^http://xvm145.jefferson.edu/progmir/

^16^http://bioinformatica.mty.itesm.mx/SurvMicro

^17^http://www.oncolnc.org

^18^https://www.tanric.org

^19^http://tcpaportal.org/

^20^https://nborcherding.shinyapps.io/TRGAted

^21^https://biit.cs.ut.ee/methsurv/

^22^http://www.cbioportal.org

## Supplementary Material

Table S1 Feature analysis of included prognostic web servers.

Table S2 Datasets and samples of prognostic web servers based on mRNA.

Table S3 Datasets and samples of prognostic web servers based on ncRNA.

Table S4 Datasets and samples of prognostic web servers based on protein.

Table S5 Datasets and samples of prognostic web servers based on DNA methylation and mutation.
