# Supplementary material for: Comprehensive Review of Web Servers and Bioinformatics Tools for Cancer Prognosis Analysis
Source: Front Oncol. 2020 Feb 5;10:68. doi: 10.3389/fonc.2020.00068 (PMC7013087; doi:10.3389/fonc.2020.00068)
Supplement: Table S1 — Feature analysis of included prognostic web servers. [file Table_1.DOCX]

**Table S1** Feature analysis of included prognostic web servers

| Web server | Strengths | Limitations | Cut-off | Running time |
| --- | --- | --- | --- | --- |
| LOGpc | More datasets  Combined datasets analysis  More follow-up information  More subgroups  Easy to operate. | No multi-gene analysis  No optimal cut-off | Upper 25%, upper 30%, upper 50%, upper 25% VS lower25%, upper 30% VS lower30%, lower25%, lower30%, lower50% | ＜30 s |
| GENT2 | Meta-survival analysis  Gene and subtype profile  Customed datasets analysis | No multi-gene analysis  No optimal cut-off  Median cut-off only | Median | ＜30 s |
| PROGgeneV2 | Pan-cancer analysis  Survival of multi-gene  Survival of gene ratio  Gene combined signature  Customized datasets analysis | No optimal cut-off | Median, 25%, 75%, mean | ＜30 s |
| SurvExpress | Multi-gene survival analysis  Response to treatment | Frequent errors during the execution of web application | - | ＞2min |
| PRECOG | Pan-caner survival analysis  Top prognostic genes | Needed to register and log in. | Median | ＞2min |
| Oncomine | Multi-omics data  Multiple functions | Needed to register and log in Less survival information. | - | ＞2min |
| PrognoScan | Multiple survival information | Only GEO data | Optimal | ＞2min |
| KM Plotter | Survival analysis based on mRNA and miRNA data  Multi-gene and pan-cancer query | Incomplete cancer types | Lower quartile, lower tertile, median, upper tertile, upper quartile, best cut-off | ＜30 s |
| GSCALite | Survival analysis based on multi-  omics data  Multiple functions | Dot graph for survival  Less follow-up information | - | ＞2min |
| UALCAN | Differential analysis  Multi-omics data  Pan-cancer analysis | Less survival information  No optimal cut-off | - | ＜30 s |
| GEPIA | Customized data,  Differential gene analysis  Similar gene analysis  Multiple gene comparison | Less follow-up information  Data mainly from TCGA | Median, quartile, custom | ＜30 s |
| CAS-viewer | Comprehensive analysis  Multi-omics data  Alternative splicing and prognosis. | Data mainly from TCGA Sometimes the web page can't be opened | - | ＞2min |
| MEXPRESS | Visual analysis of DNA methylation  Multiple clinical variables | No Kaplan-Meier plot | Median, mean, quartile | ＜30 s |
| CaPSSA | Multi-omics data survival analysis  Customized data analysis  Subtype Risk assessment | No subgroup survival analysis | Average, median | ＜30 s |
| OncoLnc | Prognostic analysis based on mRNA, miRNA and lncRNA data | Less follow-up information  No subgroup survival analysis  No multi-gene analysis | Custom | ＜30 s |
| PROGmiRV2 | Multiple miRNAs and two miRNA ratio analysis  Prognosis analysis for customized datasets | No optimal cut-off | Median, 25%, 75%, mean | ＜30 s |
| SurvMicro | Assessment of miRNA signatures for survival analysis | Sometimes the web page can't be opened | Median, quartile | ＞5min |
| TANRIC | Correlation analysis between lncRNAs and multi-omics data  Subtypes prognosis analysis  Drug sensitivity analysis  Customized data analysis | Sometimes the web page can't be opened | - | ＞2min |
| TCPAv3.0 | Protein-centric analysis  Pan-cancer analysis | Less RPPA protein data  Less follow-up information  No subgroups and multi-gene survival analysis | - | ＜30 s |
| TRGAted | More comprehensive clinical data  Subgroups survival analysis  multi-gene survival analysis | Less RPPA protein data | Quartile, tertile, median, optimal | ＞2min |
| MethSurv | Multiple survival analysis  Easy to operate | Less follow-up information | Best, median, q25, q75, mean, maxstat | ＜30 s |
| cBioPortal | Survival analysis based on DNA mutation data and CNA data  Pan-cancer analysis Multi-gene query. | Sometimes the web page can't be opened | - | ＞2min |

Note: ‘-’: related information is not displayed on the website
